# Hypoglycemic Effect of *Pleurotus citrinopileatus* and *Hericium erinaceus* Buccal Tablets on Diabetic Mice

**DOI:** 10.3390/biology14111591

**Published:** 2025-11-14

**Authors:** Zhongyi Yang, Kailu Zhang, Yan Liang, Kexin Shi, Jinqiang Ma, Juan Yu, Cunlong Lu, Aimin Liu, Xiancan Zhu

**Affiliations:** Anhui Provincial Key Laboratory of Molecular Enzymology and Mechanism of Major Metabolic Diseases, College of Life Sciences, Anhui Normal University, Wuhu 241000, China; 18895602812@163.com (Z.Y.); zhangkailu1997@163.com (K.Z.); ly10_02ly@163.com (Y.L.); 18365143488@163.com (K.S.); majinqaskx@163.com (J.M.); juanyu@ahnu.edu.cn (J.Y.); ahuo@ahnu.edu.cn (C.L.)

**Keywords:** antioxidant enzyme, blood glucose, edible mushrooms, gut microbiota, trace elements

## Abstract

Diabetes mellitus poses a global health challenge, necessitating natural adjuvants with minimal side effects. The aims of this study were to optimize the concentrations of chromium (Cr), zinc (Zn), and germanium (Ge) in the liquid fermentation media of *Pleurotus citrinopileatus* and *Hericium erinaceus* and to evaluate the hypoglycemic efficacy of buccal tablets in diabetic mice. The results showed that the optimal ion concentrations in the liquid fermentation medium were Cr 200 mg/L, Zn 200 mg/L, and Ge 50 mg/L for *P. citrinopileatus*, and Cr 200 mg/L, Zn 100 mg/L, and Ge 100 mg/L for *H. erinaceus*. After 3 weeks of administration of high-dose (6 g/kg) *P. citrinopileatus* and *H. erinaceus* buccal tablets, a 29.1% reduction in the blood glucose levels of diabetic mice was observed compared with pre-administration. High-dose tablets decreased the levels of total cholesterol, triglyceride, and low-density lipoprotein cholesterol while increasing high-density lipoprotein cholesterol. Compared with negative control, high-dose tablets increased catalase and superoxide dismutase activities by 31.2% and 34.1%, respectively. Moreover, the buccal tablets modulated the diversity and structure of the gut microbiota in mice. Relative abundance of beneficial genera (*Lactobacillus*, *Akkermansia*, *Bifidobacterium*, and *Ruminococcus*) in the high-dose group were increased, while diabetogenic taxa (*Prevotella*, *Desulfovibrio*, and *Enterococcus*) were inhibited. It is concluded that buccal tablets combining *P. citrinopileatus* and *H. erinaceus* treated with Cr, Zn, and Ge significantly ameliorated hyperglycemia, dyslipidemia, and oxidative stress, and reshaped the gut microbiota in diabetic mice, demonstrating the potential of edible mushrooms and trace elements as a natural antidiabetic therapy.

## Simple Summary

Diabetes mellitus is a chronic metabolic disorder characterized by persistent hyperglycemia that poses a global health challenge. Edible mushroom fermented foods have garnered attention for their medicinal properties, including hypoglycemic, hypolipidemic, and immunomodulatory effects. In this study, the Cr, Zn, and Ge concentrations in liquid fermentation media of *Pleurotus citrinopileatus* and *Hericium erinaceus* was optimized, *P. citrinopileatus* and *H. erinaceus* buccal tablets were prepared, and the hypoglycemic efficacy of these buccal tablets was evaluated in diabetic mice. The results showed that the edible mushroom buccal tablets ameliorated hyperglycemia, dyslipidemia, oxidative stress, and reshaped gut microbial community structure of diabetic mice. The findings could pave the way for novel, natural-based therapies for diabetes management.

## 1. Introduction

Diabetes mellitus is a chronic metabolic disorder characterized by persistent hyperglycemia resulting from defective insulin secretion, insulin resistance, or both [1]. The global prevalence of diabetes has reached alarming levels, accounting for 537 million patients and an incidence rate of 10.5% in 2021. The number of diabetes cases worldwide is projected to rise to 643 million by 2030 and 783 million by 2045, representing a 46% increase [2]. This epidemic poses significant challenges to public health systems, necessitating innovative therapeutic strategies for management and complication mitigation.

The pathogenesis of diabetes involves complex interactions between genetic predisposition, environmental factors, and lifestyle choices [3]. Chronic hyperglycemia leads to severe complications, including cardiovascular diseases, neuropathy, and nephropathy, underscoring the urgent need for effective treatments [4,5]. While conventional therapies like insulin injections and oral hypoglycemic agents (e.g., metformin) are widely used, they often come with side effects such as weight gain and gastrointestinal disturbances. Consequently, there is growing interest in natural compounds with fewer adverse effects, particularly polysaccharides derived from edible mushrooms [6].

Edible mushrooms, such as *Pleurotus* and *Hericium*, have garnered attention for their medicinal properties including hypoglycemic, hypolipidemic, and immunomodulatory effects [7,8]. Polysaccharides from these mushrooms exhibit potent bioactivities, such as enhancing insulin sensitivity and protecting pancreatic β-cells [9]. For instance, *P. citrinopileatus* Singer polysaccharides have been shown to reduce blood glucose levels by modulating gut microbiota and improving glucose metabolism [10]. Similarly, *H. erinaceus* (Bull.) Pers. powder has demonstrated efficacy in preventing weight loss and regulating lipid disorders in diabetic models [11]. However, the synergistic effects of combining these mushrooms remain unexplored, presenting a promising research avenue.

Trace elements, including chromium (Cr), zinc (Zn), and germanium (Ge), play pivotal roles in a diverse range of fundamental biological processes such as immunological response, glucose homeostasis, and insulin signaling [12]. Cr deficiency is prevalent among diabetic patients and is linked to impaired carbohydrate metabolism [13]. Zn acts as a cofactor for enzymes involved in insulin synthesis and secretion, while Ge has been reported to help normalize elevated blood glucose levels [14]. The potential interplay between these trace elements and mushroom polysaccharides could enhance their hypoglycemic efficacy, yet this area remains understudied.

The relationship between diabetes and gut microbiota is remarkably close. Gut microbiota can influence the onset and progression of diabetes by affecting physiological metabolism, immune function, and oxidative stress of the hosts [15,16]. The gut microbiota has emerged as a key regulator of glucose metabolism, with dysbiosis contributing to insulin resistance [17]. Duan et al. (2023) found that fungal polysaccharides can modulate gut microbiota composition, thereby improving metabolic outcomes [18]. Qi et al. (2024) demonstrated that *Desulfovibrio* sp., an intestinal symbiont enriched in patients with metabolic syndrome, suppresses the production of the gut hormone glucagon-like peptide 1 and promotes the occurrence of metabolic diseases such as obesity [19]. These findings highlight the potential of edible mushrooms as adjuvants in diabetes therapy.

Edible mushroom fermented foods represent a novel type of functional food. The nutritional content of the mycelia is comparable to or even higher than that of fruiting bodies, and they possess similar bioactive properties, such as antitumor, antioxidant, hypoglycemic, and hypolipidemic effects. Liquid fermentation can enhance the production of bioactive metabolites in mycelia, improving their nutritional value while imparting unique health benefits [20,21]. This study investigates the hypoglycemic effects of *P. citrinopileatus* and *H. erinaceus*. By culturing these mushrooms under optimized conditions and formulating them into buccal tablets, we aim to evaluate their efficacy in type 2 diabetic model mice. Physiological indicators such as blood glucose levels, insulin sensitivity, and gut microbiota composition were analyzed to elucidate the underlying mechanisms. The findings could pave the way for novel, natural-based therapies for diabetes management.

## 2. Materials and Methods

### 2.1. Experimental Materials

The strains of *Pleurotus citrinopileatus* (YY-02) and *Hericium erinaceus* (YH-06) were kindly provided by Wuhu Yeshulin Biotechnology Co., Ltd. (Wuhu, China).

The specific pathogen-free (SPF) grade healthy male Kunming (KM) mice, weighing 17–20 g, were supplied by Henan Skbess Biotechnology Co., Ltd. (Anyang, China). The animal production license number is SCXK (Yu) 2020-0005.

The high-sugar and high-fat diet comprised 60% fat, 20% carbohydrates, and 20% protein. The basal diet contained 12% fat, 67.4% carbohydrates, and 20.6% protein. Both diets were provided by Jiangsu Synergy Medical Biotechnology Co., Ltd. (Nanjing, China).

### 2.2. Optimization of Fermentation Medium

#### 2.2.1. Single-Factor Experiments

The effects of Cr, Zn, and Ge on the polysaccharides content of *P. citrinopileatus* and *H. erinaceus* during liquid fermentation were investigated by adding different concentrations of Cr^3+^, Zn^2+^ and Ge^4+^ into the Potato Dextrose Agar (PDA, specify the supplier) comprehensive medium. The Cr addition experiment was conducted with 6 treatments: 0, 25, 50, 100, 200, and 300 mg/L CrCl_3_ solution. The Zn addition experiment was conducted with 6 treatments: 0, 100, 200, 300, 500, and 1000 mg/L ZnSO_4_ solution. The Ge addition experiment was conducted with 6 treatments: 0, 50, 100, 200, 300, and 400 mg/L GeO_2_ solution. Each treatment was repeated 3 times.

The activated *P. citrinopileatus* and *H. erinaceus* mycelia were inoculated into PDA culture medium with different Cr^3+^, Zn^2+^, and Ge^4+^ concentrations and incubated at 25 °C with constant temperature shaking at 200 rpm for 7 days. The mycelia was filtered, dried, and the polysaccharide content was determined using the phenol sulfuric acid method [22].

#### 2.2.2. Orthogonal Experiment

Taking the polysaccharide content as the indicator, the optimal concentration intervals of the three ions obtained by the above single-factor experiment results were selected for each interval, and an orthogonal experiment of the three factors and three levels was conducted to determine Cr^3+^, Zn^2+^, and Ge^4+^ concentration ratios for the liquid fermentation media of *P. citrinopileatus* and *H. erinaceus*, respectively (Table S1).

### 2.3. Preparation and Detection of Buccal Tablets

According to the optimal ratio obtained, the strain of *P. citrinopileatus* and *H. erinaceus* was subjected to scaled-up cultivation in a 50 L fermenter at 25 °C, pH 6.5, and 150 r/min for 4 days, respectively. Subsequently, the obtained mycelia of *P. citrinopileatus* and *H. erinaceus* were filtered and dried at 60 °C before being pulverized. 30% of mycelium powders of *P. citrinopileatus* and *H. erinaceus* were mixed thoroughly with 30% xylitol, 1.5% citric acid, 1% magnesium stearate, and 37.5% filler mannitol and compressed into tablets via wet granulation method [23]. The nutritional components, microorganisms, and heavy metals of buccal tablets were measured using the methods of National Food Safety Standards. The test results are shown in Table 1, and all conformed to the National Food Safety Standards [24].

### 2.4. Mice Culture and Parameter Measurements

#### 2.4.1. Modeling of Diabetes Mice

SPF-grade healthy male KM mice were housed in the animal laboratory of Anhui Normal University under the conditions of a temperature of 23–26 °C, a relative humidity of 30–40%, and a 12 h light–dark cycle. After one week of adaptive culture, the mice were fed with the high-sugar and high-fat diet. After three weeks, following a 12 h fast with free access to water, the mice were intraperitoneally injected with streptozotocin (STZ) solution at a dosage of 120 mg/kg. Three days post-injection, fasting blood glucose (FBG) levels of the mice were measured from tail vein blood using a glucometer (GA-3, Sinocare, Changsha, China). Mice with an FBG level ≥11.1 mmol/L were considered successful modeling.

#### 2.4.2. Experimental Design

This experiment was randomly conducted in five groups with eight mice in each group, namely the normal CK group (basal diet + physiological saline), negative CK group (high-sugar and high-fat diet + physiological saline), positive CK group (high-sugar and high-fat diet + 120 mg/kg metformin), low-dose group (high-sugar and high-fat diet + 2 g/kg buccal tablets), and high-dose group (high-sugar and high-fat diet + 6 g/kg buccal tablets). The buccal tablets were ground into powder and diluted with physiological saline to formulate low-dose and high-dose tablet solutions at concentrations of 0.2 g/mL and 0.6 g/mL, respectively. The mice were gavaged daily at a volume of 0.1 mL per 10 g.

#### 2.4.3. Measurement of Body Weight, FBG, and Glucose Tolerance in Diabetic Mice

After a 12 h fast (with free access to water), the body weight of mice was weighed weekly. The FBG levels were measured weekly by tail vein blood sampling.

For the oral glucose tolerance test, performed before the final administration, mice were fasted for 12 h. After 30 min of administration, each group was given a dose of 2.0 g/kg glucose by gavage. FBG levels were measured from the tail vein at 0, 30, 60, and 120 min.

#### 2.4.4. Determination of Blood Lipids

The mouse serum was collected, and the levels of total cholesterol (TC), triglyceride (TG), low-density lipoprotein cholesterol (LDL-C), and high-density lipoprotein cholesterol (HDL-C) were measured using commercial kits (Beijing Labgic Technology Co., Ltd., Beijing, China), according to the manufacturer’s protocol.

#### 2.4.5. Measurement of Antioxidant Enzymes

Catalase (CAT) and superoxide dismutase (SOD) activities in mouse serum were determined using commercial kits (Beijing Labgic Technology Co., Ltd., Beijing, China), according to the manufacturer’s protocol.

#### 2.4.6. NovaSeq Sequencing

After euthanasia, mouse abdominal cavities were dissected under sterile environment. The colons were excised with sterile scissors and the samples were placed into 2 mL sterile EP tubes and stored at −80 °C. DNA was extracted using a DNA extraction kit according to the manufacturer’s instructions. Additionally, DNA quality was measured by 0.8% agarose gel electrophoresis, while DNA content was measured by ultraviolet spectrophotometer (Nanodrop NC2000, Thermo Scientific, Waltham, MA, USA).

The V3-V4 region of the 16S rDNA gene was amplified via PCR using primers of 338F (ACTCCTACGGGAGGCAGCA) and 806R (GGACTACHVGGGTWTCTAAT) [39]. According to primary electrophoresis evaluation, Quant-iTPicoGreen dsDNA Assay Kit (Invitrogen, Camarillo, CA, USA) was applied in quantifying PCR amplification product fluorescence. Afterwards, purified DNA samples were integrated for sequencing using Illumina NovaSeq 6000 platform (Illumina, San Diego, CA, USA) and NovaSeq 6000 S4 Reagent Kit V1.5 (paired-end, 300 cycles, 2 × 250 bp) at Suzhou Panomics Biomedical Technology Co., Ltd., China (Suzhou, China).

#### 2.4.7. Sequencing Data Analysis

The raw data were processed using QIIME2 2019.4 [40]. Subsequently, quality control, denoising, splicing, and chimera removal were performed on the clean reads to obtain the optimal sequences [41]. A total of 42,335 sequences/samples were rarefied. On the basis of the optimized sequences, amplicon sequence variants (ASVs) clustering analysis and species taxonomic annotation were carried out. Alpha and beta diversity metrics were performed based on the ASV clustering results. By adopting classify-sklearn naive Bayes taxonomy classifier, this work assigned taxonomies to ASVs through the feature-classifier plugin based on Silva v132 99% operational taxonomic units reference sequences [42]. Moreover, using R package (v4.1.0), inconsistency in the composition of bacterial communities was illustrated by non-metric multidimensional scaling (NMDS) based on Bray–Curtis distance. KEGG pathways were enriched by PICRUSt2 method [43].

### 2.5. Statistical Analysis

IBM SPSS22.0 (SPSS Inc., Armonk, NY, USA) and R (v4.1.0) were performed for statistical analysis. Before statistical analysis, the data were transformed logarithmically; as a result, the variables were as close to a normal distribution as possible. Duncan’s test was utilized for post hoc multi-comparison of means from diverse apparent effects (F-value). *p* < 0.05 stood for statistical significance. Analysis of variance (ANOVA) is used for orthogonal experiments. The K value represents the average value of a variable at various levels. The R value represents the difference between the maximum K value and the minimum K value of a variable. Pearson correlation analysis is used for determining coefficients of correlation among mice blood parameters and microbial communities.

## 3. Results

### 3.1. Optimization of Fermentation Conditions in P. citrinopileatus and H. erinaceus

By single-factor experiments, the optimal concentration ranges for Cr^3+^, Zn^2+^, and Ge^4+^ components were identified using polysaccharide content as the primary evaluation criterion (Table S1, Figures S1 and S2).

The range R reflects the extent of the influence of experimental factors on the outcomes. A larger value of R indicates a stronger influence. According to the results of R values, the influence of ions on polysaccharide yield during liquid fermentation of *P. citrinopileatus* was ranked as Zn^2+^ > Ge^4+^ > Cr^3+^ (Table 2). The optimal ion concentration proportion for the liquid fermentation medium of *P. citrinopileatus* was Cr^3+^ 200 mg/L, Zn^2+^ 200 mg/L, and Ge^4+^ 50 mg/L, which yielded the maximum polysaccharide content.

The influence of the three ions on the polysaccharide yield of *H. erinaceus* during liquid fermentation, ranked in descending order, is Zn^2+^ > Ge^4+^ > Cr^3+^ (Table 2). Upon analyzing the results of the K values, the ideal combination comprises Cr^3+^ at 200 mg/L, Zn^2+^ at 100 mg/L, and Ge^4+^ at 100 mg/L, at which *H. erinaceus* attains a peak polysaccharide content.

### 3.2. Effect of Buccal Tablets on Body Weight of Diabetic Mice

**Figure 1:**
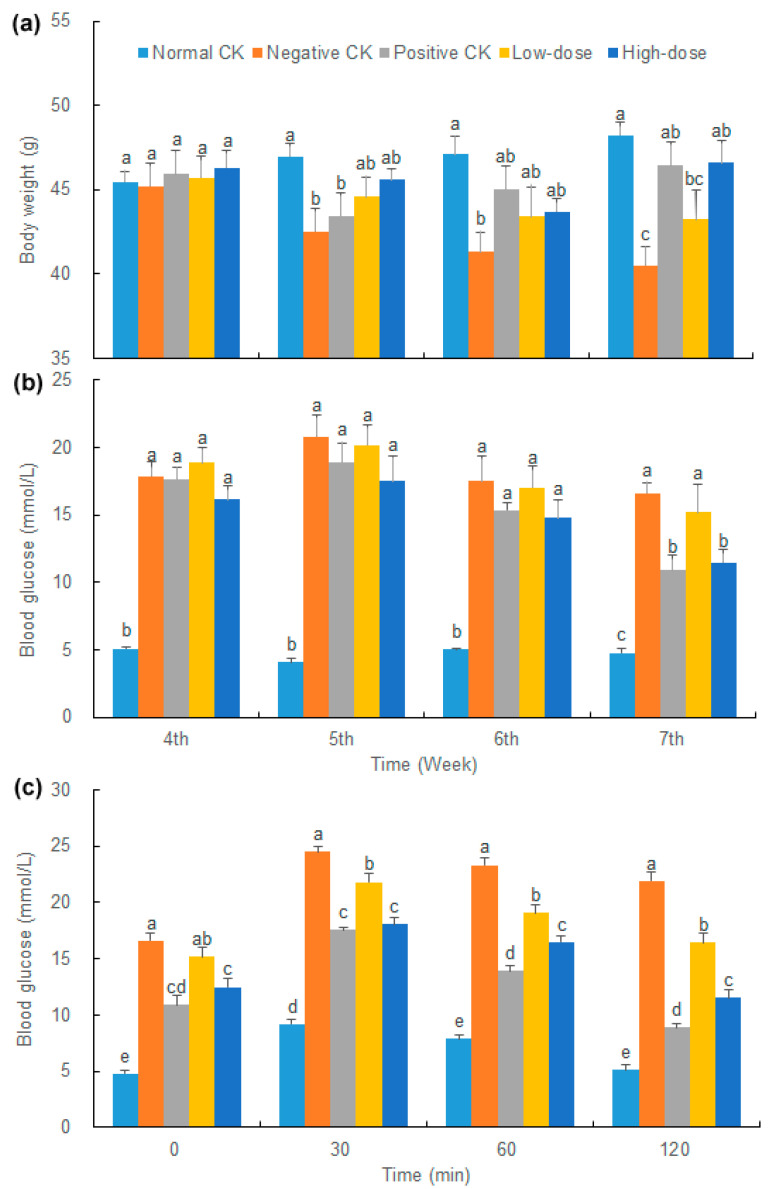
Effect of buccal tablets on (**a**) body weight, (**b**) blood glucose, and (**c**) blood glucose tolerance in serum of diabetic mice. The error bars represent standard errors. Different letters indicate significant difference (*p* < 0.05) (*n* = 8).

After three weeks of intragastric administration, the body weights of the negative CK and low-dose mice were decreased by 10.39% and 5.37%, respectively, while the normal CK, positive CK, and high-dose mice were increased by 6.05%, 1.115% and 0.685%, respectively (Figure 1a). By the seventh week, the high-dose mice significantly increased their body weight compared with the negative CK group with no significant difference from the normal CK group.

### 3.3. Effect of Buccal Tablets on Blood Glucose and Lipids of Diabetic Mice

The blood glucose levels of the normal CK mice were consistently and significantly lower than those of each diabetic group (Figure 1b). Prior to modeling, FBG levels of the mice in all diabetic groups exceeded 11.1 mmol/L, indicating the successful establishment of the STZ-induced diabetic mouse model. After three weeks of treatment, FBG levels of mice in the positive control group and the high-dose group decreased significantly by 38.36% and 29.10%, respectively. The FBG level in the high-dose group was close to that in the positive control group. Compared with the negative control group, the FBG of mice in the high-dose group decreased by 30.95%.

After oral glucose administration for 30 min, the blood glucose of mice in each group reached the highest level (Figure 1c). Subsequently, blood glucose declined, with the rate of decrease in the high-dose group being similar to that in the positive CK group, suggesting that the buccal tablets had a certain improving effect on the glucose metabolism and enhanced the glucose tolerance in the diabetic model mice.

**Figure 2:**
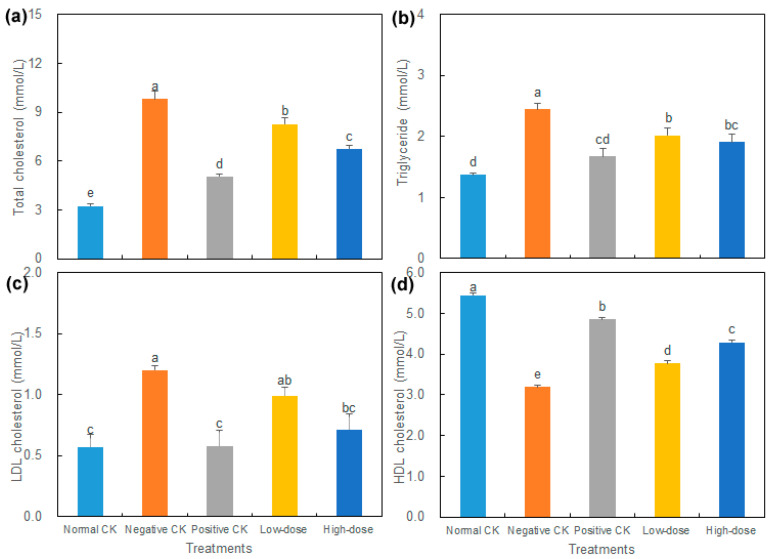
Effect of buccal tablets on lipid contents in serum of diabetic mice. (**a**) Total cholesterol. (**b**) Triglyceride. (**c**) Low-density lipoprotein (LDL) cholesterol. (**d**) High-density lipoprotein (HDL) cholesterol. The error bars represent standard errors. Different letters indicate significant difference (*p* < 0.05) (*n* = 8).

The levels of TC, TG, and LDL-C in the diabetic mice groups were all higher than those in the normal CK group, while the level of HDL-C was lower, which indicates that the blood lipid metabolism of STZ-induced diabetic mice was abnormal (Figure 2). The TC, TG, and LDL-C levels in both the positive CK group and the high-dose group were markedly reduced, while the HDL-C level was significantly increased compared to the negative CK. Compared with the normal CK, the TC, TG, and LDL-C levels in the high-dose group were increased by 108.14%, 39.4%, and 25%, respectively, while the HDL-C level was decreased by 21.12%.

### 3.4. Effect of Buccal Tablets on Antioxidant Enzymes of Diabetic Mice

**Figure 3:**
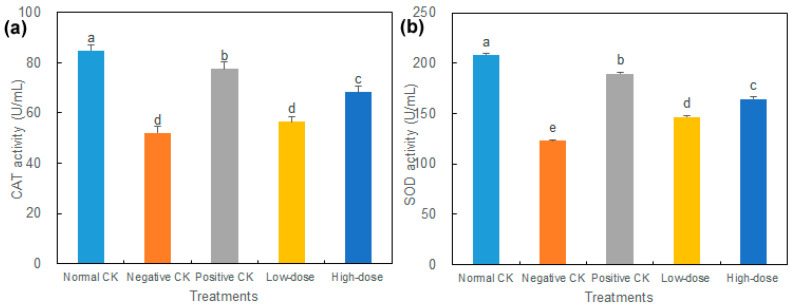
Effect of buccal tablets on antioxidant enzyme activities in serum of diabetic mice. (**a**) Catalase (CAT) activity. (**b**) Superoxide dismutase (SOD) activity. The error bars represent standard errors. Different letters indicate significant difference (*p* < 0.05) (*n* = 8).

Compared with the normal CK, a decline in the activities of CAT and SOD was observed in all diabetic mice (Figure 3). The high-dose mice decreased CAT and SOD activities by 19.42% and 21.02%, respectively, and the low-dose mice decreased by 33.27% and 29.55%, respectively, compared to the normal CK. The activities of CAT and SOD in the high-dose group were significantly increased by 31.23% and 34.14%, respectively, compared with the negative CK. The SOD activity in the low-dose group was increased by 19.65% compared to the negative CK.

### 3.5. Effect of Buccal Tablets on Microbial Community Diversity

**Figure 4:**
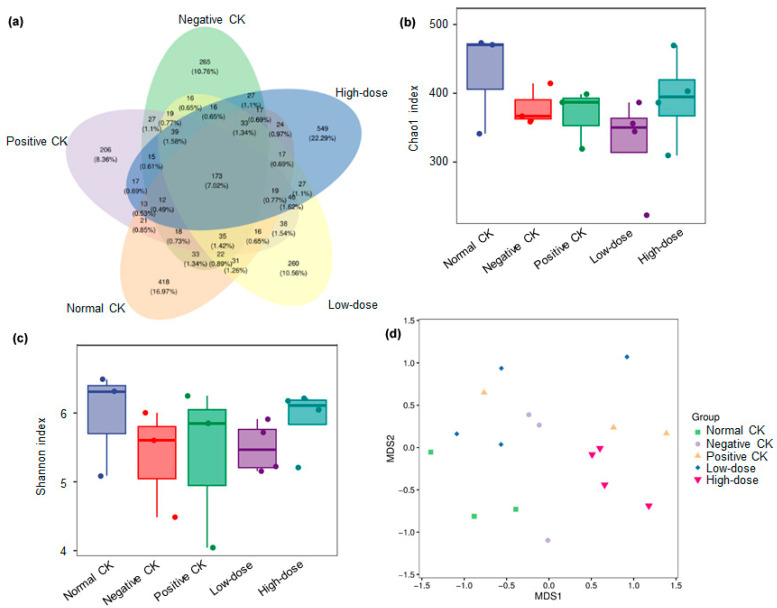
Effect of buccal tablets on microbial community diversity of diabetic mice. (**a**) Venn diagram of amplicon sequence variants (ASVs) number. (**b**) Chao1 index. (**c**) Shan-non index. (**d**) Microbial community composition indicated by non-metric multidimen-sional scaling (NMDS) plots.

A total of 2463 ASVs were detected across all 17 samples, with 173 ASVs shared among the samples of the five treatment groups (Figure 4a). The high-dose group displayed the highest number of unique ASVs, following by the normal CK, negative CK, and low-dose group, and the positive CK were the lowest.

The alpha diversity (Chao1 and Shannon index) of microbial communities was not significantly different among the treatments (Figure 4b,c). However, a significant difference in the beta diversity (NMDS) was observed among the treatments (Figure 4d).

### 3.6. Effect of Buccal Tablets on Microbial Community Abundance

**Figure 5:**
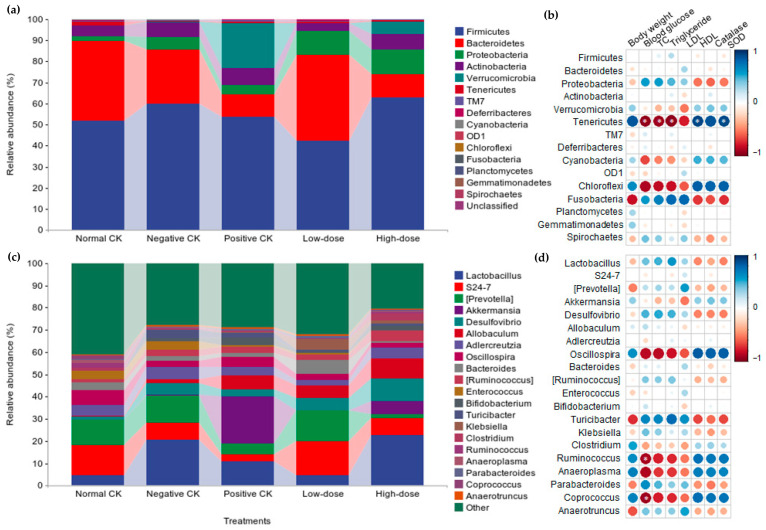
Effect of buccal tablets on microbial community abundance of diabetic mice. (**a**) Relative abundance of phyla. (**b**) Relationship between relative abundance of phyla and body weight, blood glucose, total cholesterol (TC), triglyceride, low-density lipoprotein cholesterol (LDL), high-density lipoprotein cholesterol (HDL), catalase, and superoxide dismutase (SOD). (**c**) Relative abundance of top 20 genus. (**d**) Relationship between relative abundance of genus and body weight, blood glucose, TC, triglyceride, LDL, HDL, catalase, and SOD. * indicate significant difference (*p* < 0.05).

All ASVs were classified into 17 phyla, 39 classes, 65 orders, 109 families, and 182 genera. The relative abundances of the top 20 bacterial phyla, classes, orders, families, and genera are shown in Figure 5 and Figure S3. Typically, Firmicutes, Clostridia, Clostridiales, Lactobacillaceae, and Lactobacillus were the predominant phylum, class, order, family, and genus, with relative abundances of 54.1%, 28.75%, 28.75%, 12.75%, and 12.70%, respectively.

At the phyla level, the high-dose mice had higher relative abundance of Firmicutes, Proteobacteria, Verrucomicrobia, and Tenericutes, while having lower Bacteroidetes and Actinobacteria compared with the negative CK group (Figure 5a). Moreover, the relative abundance of Tenericutes was negatively associated with blood glucose, TC, and TG while being positively associated with HDL-C and SOD.

At the genus level, the high-dose mice had higher relative abundance of *Lactobacillus*, *S24-7*, *Akkermansia*, *Desulfovibrio*, *Allobaculum*, *Ruminococcus*, *Bifidobacterium*, *Klebsiella*, and *Clostridium*, while having lower *Prevotella*, *Adlercreutzia*, *Oscillospira*, *Bacteroides*, *Enterococcus*, and *Turicibacter* compared with the negative CK group (Figure 5c). Furthermore, blood glucose content was negatively related with relative abundance of *Ruminococcus* and *Coprococcus*.

## 4. Discussion

**Figure 6:**
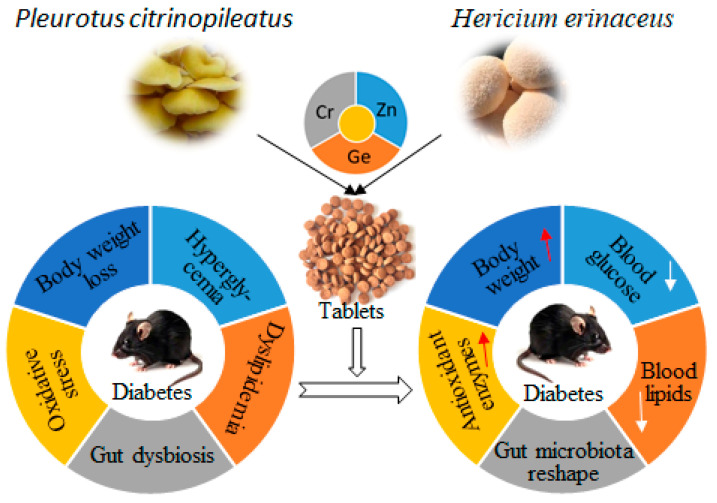
The hypoglycemic potential of buccal tablets of *Pleurotus citrinopileatus* and *Hericium erinaceus* in diabetic mice. Red and white arrows represent the increase or decrease, respectively.

This study demonstrated that buccal tablets of *P. citrinopileatus* and *H. erinaceus* significantly ameliorate hyperglycemia, dyslipidemia, oxidative stress, and gut dysbiosis in STZ-induced diabetic mice (Figure 6). The high-dose tablets (6 g/kg) exhibited efficacy comparable to metformin, highlighting their potential as a natural adjuvant therapy for diabetes management.

Metal ions influence both the growth of edible mushrooms and the production of bioactive substances in their cells. In this study, liquid culture with the addition of appropriate concentrations of metal ions can significantly increase the intracellular polysaccharide content in mycelia of *P. citrinopileatus* and *H. erinaceus*. The result was consistent with the previous studies in that metal ions increased polysaccharide in *P. djamor*, *Cordyceps military*, and *Auricularia auricula* [44,45,46]. Liu (2023) found that after Cr^3+^ enters the mycelial cells of *C. militaris*, the -COOH and -OH functional groups in the polysaccharide structure of *C. militaris* readily bind with Cr^3+^ to form chromium-enriched polysaccharides [45]. By enhancing the activity of glucose tolerance factor, Cr^3+^ further elevates insulin levels in human blood, promotes cellular absorption of amino acids and blood glucose, inhibits the breakdown of adipose tissue, and consequently reduces blood sugar levels [47,48]. Edible mushrooms possess a strong zinc-accumulating ability, enabling them to incorporate inorganic zinc into macromolecular bioactive substances to form organic zinc polysaccharides, thereby enhancing the antioxidant and immune-boosting functions of Zn [14,49]. A low concentration of Ge^4+^ promotes the synthesis of polysaccharides and other chemical components in edible mushrooms mycelia, while a high concentration can be inhibitory.

In this study, the body weight of diabetic mice was decreased compared with normal mice. Body weight serves as a crucial indicator in assessing the health condition of an organism. In diabetic patients, weight loss is potentially attributable to the diminished absorption efficiency of proteins as well as carbohydrates within the body [50]. However, diabetic mice gavaged with high-dose buccal tablets of *P. citrinopileatus* and *H. erinaceus* showed a prevention of the weight loss, with body weight nearly matching that of the normal mice by the end of the study. The results indicate that *P. citrinopileatus* and *H. erinaceus* buccal tablets alleviated the weight loss symptoms caused by diabetes, which was similar to the findings of previous studies [23,51,52]. Moreover, the lack of a significant effect in the low-dose group suggests that a sufficient dosage is necessary for efficacy.

Blood glucose levels reflect the body’s ability to utilize energy. Stable blood glucose can continuously supply energy to the body, and hyperglycemia is a typical characteristic of diabetes [53]. In the present study, high-dose treatment reduced FBG compared with negative CK and improved glucose clearance during tolerance tests, mirroring metformin effects, which suggests enhanced hepatic glucose utilization and peripheral insulin sensitivity. Cai et al. (2020) demonstrated that after 4 weeks of continuous oral administration of *H. erinaceus* polysaccharide, it reduced the FBG levels and enhanced the glucose tolerance in STZ-induced diabetic rats, thereby exerting potent hypoglycemic activity [23].

Hyperlipidemia is characterized by high levels of TC, TG, and LDL-C and a low level of HDL-C [23]. In this study, the high-dose treatment group had a significant reduction in TC, TG, and LDL-C, coupled with elevated HDL-C, indicating restored lipid homeostasis. Tao et al. (2025) also found *P. citrinopileatus* polysaccharides decreased the levels of TC, TG, LDL-C, and nonestesterified fatty acid [52]. Further quantitative real-time PCR revealed that administration of *P. citrinopileatus* polysaccharides increased the relative expressions of lipolysis related genes (*AMPKα* and *PPARα*) and decreased the expressions of lipid biosynthesis genes (*PPARγ*, *FAS* and *SERBP-1c*). Moreover, fungal β-glucans likely contributed by inhibiting cholesterol synthesis and promoting fecal bile acid excretion [8].

Increased oxidative stress and imbalance in antioxidant enzymes are key features in the occurrence and development of diabetes and its complications [51,54]. Our study showed that administration of *P. citrinopileatus* and *H. erinaceus* tablets increased serum CAT and SOD activities, which suggests that polysaccharide–metal complexes may directly scavenge free radicals by upregulating endogenous antioxidant enzymes. It is well known that edible mushroom polysaccharides have notable activities in scavenging free radicals, inhibiting lipid peroxidation, and alleviating oxidative stress [53,55]. Furthermore, this antioxidant capacity likely stems from polysaccharide-mediated Nrf2 pathway activation [9], mitigating oxidative damage implicated in diabetic complications [4].

In this study, the buccal tablets induced significant shifts in gut microbial community structure (beta-diversity), despite unchanged alpha-diversity. Notably, the high-dose group significantly reshaped gut microbial composition. Gut microbiota are capable of decomposing carbohydrates to produce short-chain fatty acids, such as acetate, propionate, butyrate, and lactate [53]. Bacteroidetes are major producers of acetic acid and propionic acid, while Firmicutes are main producers of butyric acid. Both Firmicutes and Bacteroidetes are the absolutely dominant bacterial phyla in the gut of both diabetic mice and healthy mice [52,56,57]. Acetate contributes to stabilizing the gut environment, while butyrate can increase the level of Lactobacillus and reduce the content of *Escherichia coli* in the gut, thus maintaining gut health [58]. Verrucomicrobia, abundant in healthy individuals, decompose polysaccharides, such as mucopolysaccharides and cellulose, to provide energy and nutrients. They also can generate short-chain fatty acids to modulate the immune system and maintain gut health [59]. Moreover, our study showed negative associations between abundance of Tenericutes and blood glucose, TC, and TG while showing positive relations to HDL-C and SOD, which indicates microbiota-mediated metabolic improvements [16].

In the present study, administration of *P. citrinopileatus* and *H. erinaceus* tablets increased beneficial genera (*Lactobacillus*, *Akkermansia*, *Bifidobacterium*, and *Ruminococcus*) and suppressed diabetogenic genera (*Prevotella*, *Desulfovibrio*, and *Enterococcus*). Sun et al. (2024) also found that polysaccharides of edible mushrooms increased the prevalence of *Akkermansia*, *Bifidobacterium*, and *Ruminococcus* [55]. *Lactobacillus* can prevent the colonization of pathogenic bacteria by competing for nutrients and adhesion sites, maintaining the balance of the microflora, improving digestive function, and enhancing immunity [52,60]. *Akkermansia*, as one of the major bacterial genera in the composition of the gut microbiota, plays a key role in maintaining metabolic homeostasis, promoting gut barrier integrity and GLP-1 secretion [55,61]. Ansaldo et al. (2019) revealed that *Akkermansia* can specifically induce T cells and dendritic cells, thereby regulating metabolism and adaptive immunity [62]. *Bifidobacterium* can enhance insulin sensitivity [63]. *Desulfovibrio* is critical, as this genus inhibits GLP-1 production and exacerbates metabolic dysfunction [19]. Furthermore, the relative abundance of *Ruminococcus* and *Coprococcus* negatively correlated with blood glucose, suggesting their role in short-chain fatty acids production and gluconeogenesis regulation [17,64].

## 5. Conclusions

In summary, this study optimized the Cr, Zn, and Ge concentrations for the liquid fermentation medium of *P. citrinopileatus* and *H. erinaceus* and validated the hypoglycemic potential of *P. citrinopileatus* and *H. erinaceus* buccal tablets, mediated through multi-targeted effects on glucose/lipid metabolism, oxidative stress, and gut microbiota. These findings underscore the potential of edible mushrooms as a promising natural adjuvant strategy for managing diabetes and its associated metabolic complications. Future work should explore clinical efficacy in diabetic patients and elucidate specific polysaccharide-metal interactions.

## Figures and Tables

**Table 1:** Nutritional composition, microbial limit, and heavy metal contents of buccal tablets.

Inspection Items	Inspection Findings	Testing Criterion	Stipulated by the Standard	Determination Outcome
Energy content (kJ/100 g)	1748	GB/Z21922-2008 [25]	/	/
Protein content (g/100 g)	14.2	GB5009.5-2016 [26]	/	/
Lipid content (g/100 g)	5.2	GB5009.6-2016 [27]	/	/
Carbohydrate composition (g/100 g)	77.3	GB/Z21922-2008	/	/
Sodium content (mg/100 g)	3.06	GB5009.91-2017 [28]	/	/
Total aerobic plate count (CFU/g)	220	GB4789.2-2022 [29]	≤3 × 10^4^	Compliant
Coliform group (MPN/g)	<0.3	GB4789.3-2016 [30]	≤0.92	Compliant
Molds and yeasts (CFU/g)	<10	GB4789.15-2016 [31]	≤50	Compliant
*Salmonella* (/25 g)	Undetected	GB4789.4-2016 [32]	≤0	Compliant
*Staphylococcus aureus* (/25 g)	Undetected	GB4789.10-2016 [33]	≤0	Compliant
Lead (mg/kg)	0.095	GB5009.12-2017 [34]	≤2.0	Compliant
Total arsenic (mg/kg)	Undetected (<0.01)	GB5009.11-2014 [35]	≤1.0	Compliant
Total mercury (mg/kg)	Undetected (<0.003)	GB5009.17-2021 [36]	≤0.3	Compliant
Chromium (mg/kg)	Undetected (<0.01)	GB5009.123-2014 [37]	≤0.5	Compliant
Zinc (mg/kg)	440	GB5009.14-2017 [38]	/	/

**Table 2:** Orthogonal test design results for liquid fermentation of *Pleurotus citrinopileatus* and *Hericium erinaceus*.

Protocol	Ion Content			Polysaccharide Content (mg/g)	
	Cr^3+^	Zn^2+^	Ge^4+^	*P. citrinopileatus*	*H. erinaceus*
1	1	1	1	19.487	26.626
2	1	2	3	16.686	13.375
3	1	3	2	23.358	11.159
4	2	1	2	26.936	21.764
5	2	2	1	12.487	19.720
6	2	3	3	25.671	18.729
7	3	1	3	21.100	26.005
8	3	2	2	15.772	19.220
9	3	3	1	14.358	23.419
*P*. *citrinopileatus*					
K1	59.531	67.522	46.332		
K2	65.094	44.945	66.066		
K3	51.229	63.387	63.456		
R	13.864	22.577	19.734		
*H. erinaceus*					
K1	51.161	74.395	69.765		
K2	60.213	52.316	52.143		
K3	68.644	53.307	58.109		
R	17.484	22.079	17.622		

## Data Availability

The original contributions presented in the study are included in the article.

## Supplementary Materials

The following supporting information can be downloaded at https://www.mdpi.com/article/10.3390/biology14111591/s1. Figure S1: Effects of different concentrations of Cr, Zn, and Ge on the polysaccharide content of *Pleurotus citrinopileatus*; Figure S2: Effects of different concentrations of Cr, Zn, and Ge on the polysaccharide content of *Hericium erinaceus*; Figure S3: Effect of buccal tablets on relative abundance of top 20 class (a), order (b), and family (c) of diabetic mice; Table S1: Orthogonal test factor level for optimization of fermentation conditions of *Pleurotus citrinopileatus* and *Hericium erinaceus*.
