# Supplementary material for: Hypoglycemic Effect of Pleurotus citrinopileatus and Hericium erinaceus Buccal Tablets on Diabetic Mice
Source: Biology (Basel). 2025 Nov 14;14(11):1591. doi: 10.3390/biology14111591 (PMC12649960; doi:10.3390/biology14111591)
Supplement: Supplementary file 1 [file biology-14-01591-s001.zip › biology-3872576-supplementary.pdf]

Table S1 Orthogonal test factor level for optimization of fermentation conditions of *Pleurotus citrinopileatus* and *Hericium erinaceus*

	Cr^{3+} (mg/L)		Zn^{2+} (mg/L)		Ge^{4+} (mg/L)	
	P. citrinopileatus	*H. erinaceus*	*P. citrinopileatus*	*H. erinaceus*	*P. citrinopileatus*	*H. erinaceus*
1	100	50	200	100	0	100
2	200	100	300	200	50	200
3	300	200	500	300	100	300

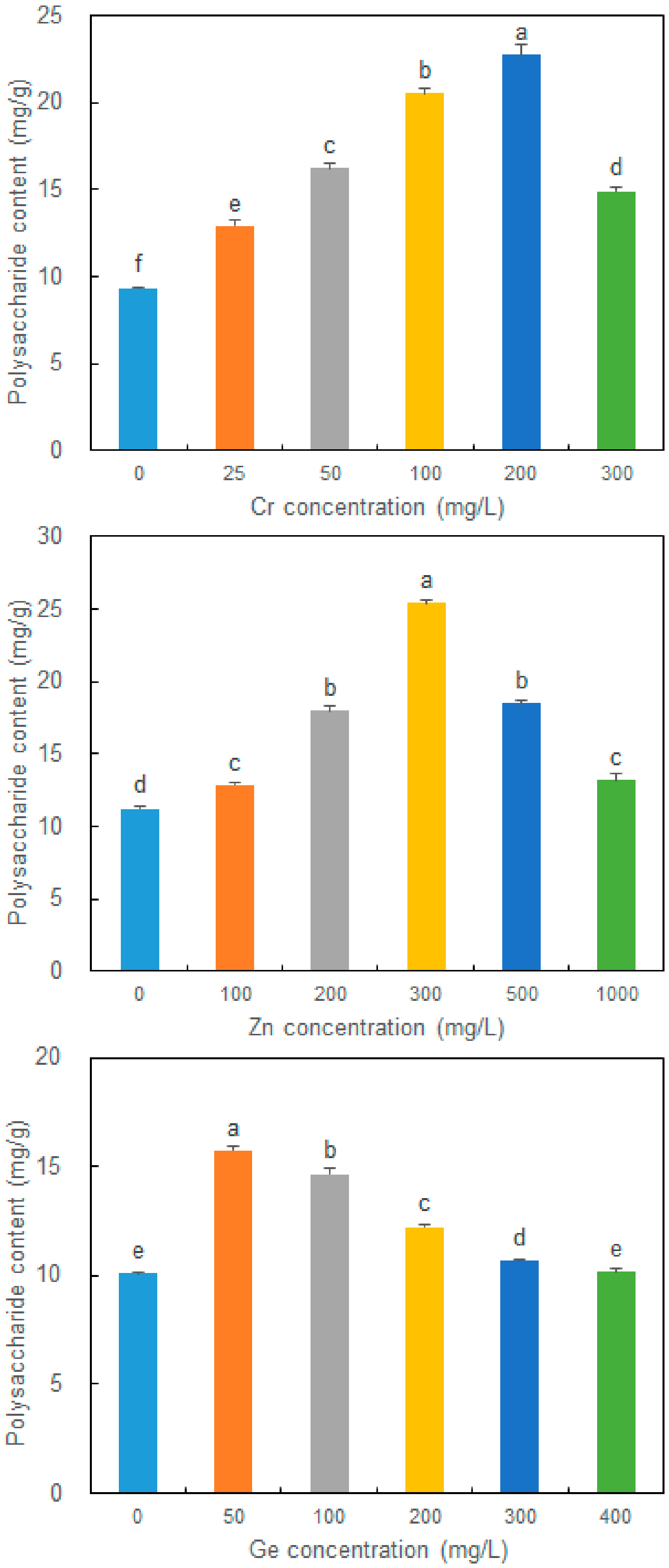

Figure S1 Effects of different concentrations of Cr, Zn, and Ge on the polysaccharide content of *Pleurotus citrinopileatus*. Different letters indicate significant difference ($p < 0.05$) ($n = 8$).

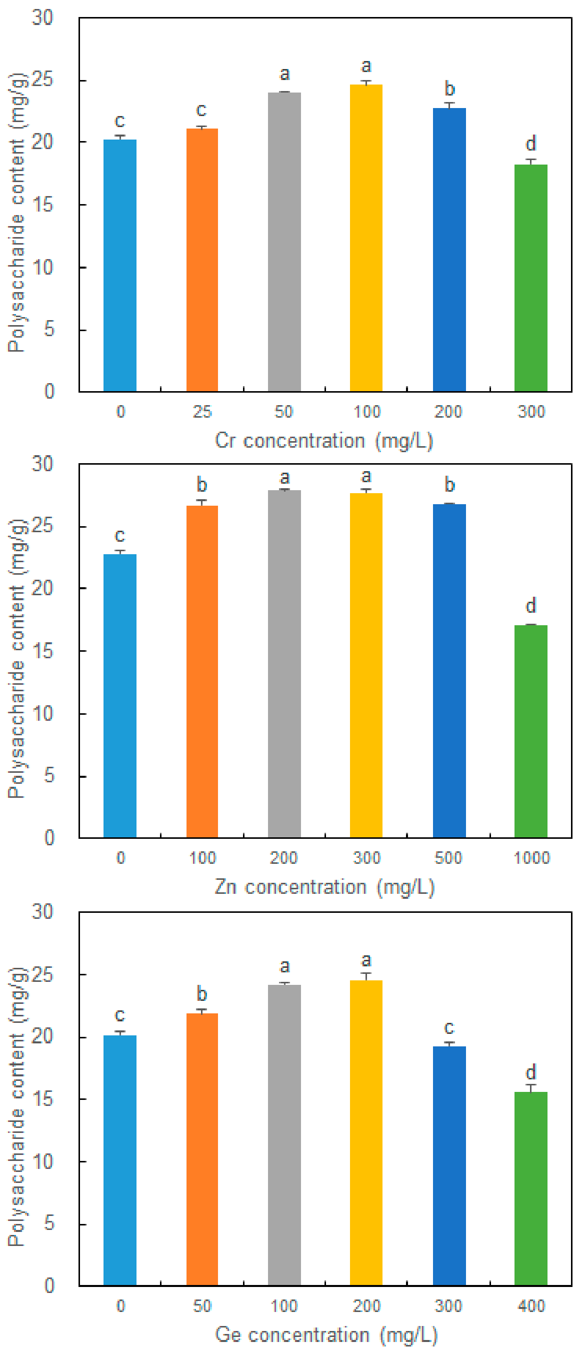

Figure S2 Effects of different concentrations of Cr, Zn, and Ge on the polysaccharide content of *Hericium erinaceus*. Different letters indicate significant difference ($p < 0.05$) (n = 8).

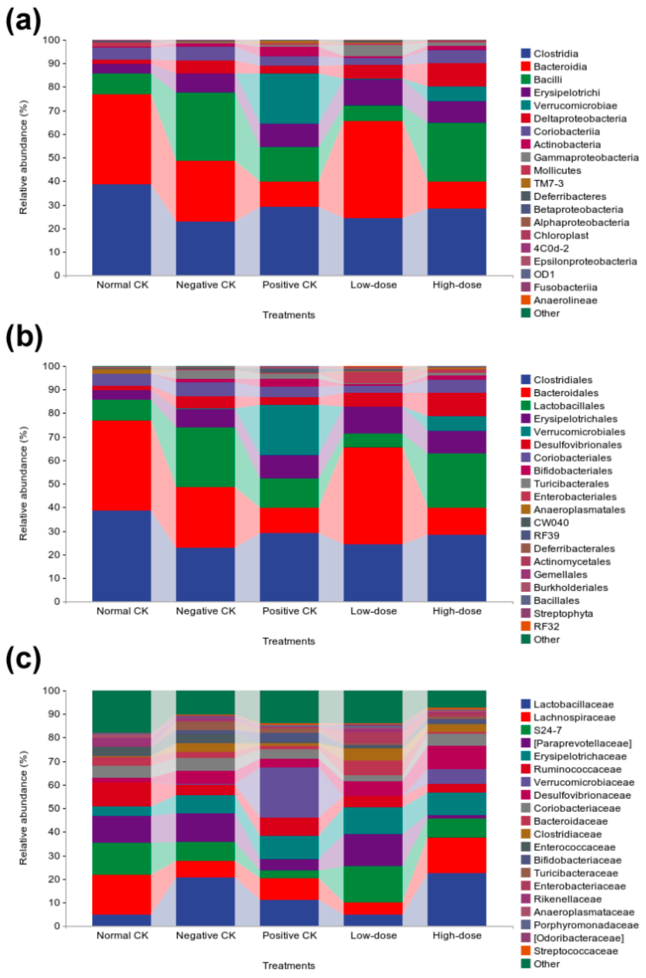

Figure S3 Effect of buccal tablets on relative abundance of top 20 class (a), order (b), and family (c) of diabetic mice
